# The Clinical Utilisation of Respiratory Elastance Software (CURE Soft): a bedside software for real-time respiratory mechanics monitoring and mechanical ventilation management

**DOI:** 10.1186/1475-925X-13-140

**Published:** 2014-09-30

**Authors:** Akos Szlavecz, Yeong Shiong Chiew, Daniel Redmond, Alex Beatson, Daniel Glassenbury, Simon Corbett, Vincent Major, Christopher Pretty, Geoffrey M Shaw, Balazs Benyo, Thomas Desaive, J Geoffrey Chase

**Affiliations:** Department of Control Engineering and Information, Budapest University of Technology and Economics, Budapest, Hungary; Centre for BioEngineering, University of Canterbury, Canterbury, New Zealand; Department of Intensive Care, Christchurch Hospital, Canterbury, New Zealand; GIGA Cardiovascular Science, University of Liege, Liege, Belgium

**Keywords:** Mechanical Ventilation, Software, Respiratory Mechanics, Monitoring, Decision Making, Positive End-Expiratory Pressure (PEEP)

## Abstract

**Background:**

Real-time patient respiratory mechanics estimation can be used to guide mechanical ventilation settings, particularly, positive end-expiratory pressure (PEEP). This work presents a software, Clinical Utilisation of Respiratory Elastance (CURE Soft), using a time-varying respiratory elastance model to offer this ability to aid in mechanical ventilation treatment.

**Implementation:**

CURE Soft is a desktop application developed in JAVA. It has two modes of operation, 1) Online real-time monitoring decision support and, 2) Offline for user education purposes, auditing, or reviewing patient care. The CURE Soft has been tested in mechanically ventilated patients with respiratory failure. The clinical protocol, software testing and use of the data were approved by the New Zealand Southern Regional Ethics Committee.

**Results and discussion:**

Using CURE Soft, patient’s respiratory mechanics response to treatment and clinical protocol were monitored. Results showed that the patient’s respiratory elastance (*Stiffness*) changed with the use of muscle relaxants, and responded differently to ventilator settings. This information can be used to guide mechanical ventilation therapy and titrate optimal ventilator PEEP.

**Conclusion:**

CURE Soft enables real-time calculation of model-based respiratory mechanics for mechanically ventilated patients. Results showed that the system is able to provide detailed, previously unavailable information on patient-specific respiratory mechanics and response to therapy in real-time. The additional insight available to clinicians provides the potential for improved decision-making, and thus improved patient care and outcomes.

**Electronic supplementary material:**

The online version of this article (doi:10.1186/1475-925X-13-140) contains supplementary material, which is available to authorized users.

## Background

Patients with respiratory failure require mechanical ventilation (MV) for breathing support. In particular, patients with acute respiratory distress syndrome (ARDS) are mechanically ventilated to maintain alveoli recruitment allowing better oxygenation, reducing their work of breathing and aiding recovery. The causes of respiratory failure are often different between patients, and more importantly, responses to treatment can be highly patient-specific. Thus, there is a patient-specific optimal ventilator setting that, if determined, could potentially improve patient care and outcome [1, 2].

MV supports patients’ work of breathing until the underlying disease is resolved. However, there is little or no consensus to select patient-specific optimal ventilator settings with several iterations of the ARDSNet tables that several, but not all, clinicians use as a basis [3, 4]. Clinicians often resort to experience, intuition or generalised approaches to select MV settings [5–9]. Thus, a great deal of research seeks to aid clinicians in optimising patient-specific ventilator setting.

In one case, a model-based approach is being investigated to select best positive end expiratory pressure (PEEP) [2]. PEEP is the additional pressure applied at the end of expiration to recruit and retain collapsed alveoli at expiration [10]. Studies have revealed that higher PEEP can be beneficial for ARDS patients [6], but high PEEP also risks barotrauma and detrimental effects on healthy and mildly injured alveoli [11, 12]. Such damage negates positive effects and further complicates the patient condition and care. Thus, determining and setting optimal patient-specific PEEP during MV must balance risk and reward [1, 2].

One of the patient-specific PEEP selection method is through setting PEEP between lower inflection point (LIP) and upper inflection point (UIP) of the static compliance curve [13]. However, the patient-specific static compliance curve is highly variable with the LIP or UIP not identifiable during tidal ventilation. Thus, the application of this method remains limited because it requires additional protocol and interrupting care. Another patient-specific PEEP selection method was through monitoring respiratory mechanics during PEEP changes. In particular, it was found in studies that patient-specific PEEP set to minimal elastance (or maximum compliance) can improve oxygenation and balance lung recruitment and avoid overdistension [2, 14–18].

This manuscript presents a software program, Clinical Utilisation of Respiratory Elastance Software (CURE Soft), equipped with a respiratory mechanics model to calculate patient-specific respiratory mechanics (respiratory elastance and resistance) from real-time data provided by the ventilator. CURE Soft is also embedded with a decision support system to titrate a patient-specific ventilator PEEP level at the bedside, using the calculated patient-specific respiratory mechanics. This software is developed in JAVA, and can be used in real-time connected to a Puritan Bennett 840 ventilator, or similar device. It also has an offline setting that allows re-simulation of the clinical situation for training purposes. CURE Soft is tested in a clinical settings and the findings are presented here for evaluation.

## Implementation

**Figure 1:**
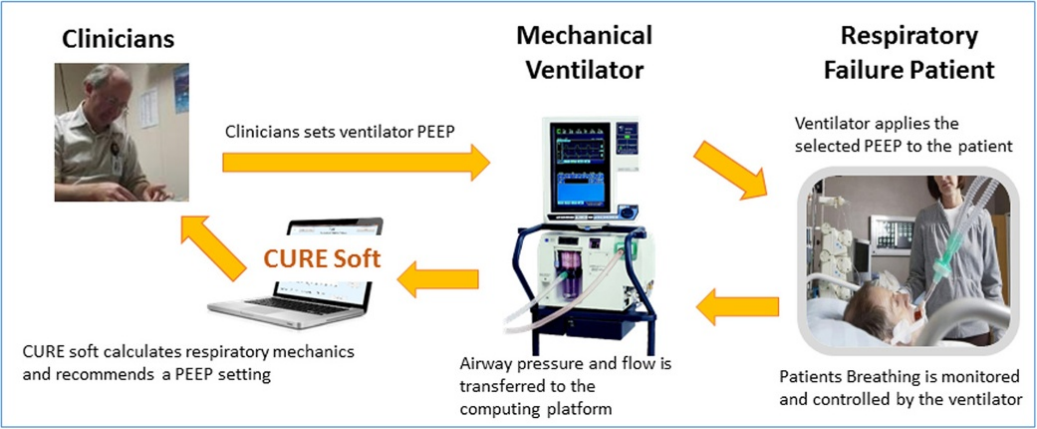
**Schematic of CURE Soft implementation in the ICU.**

The aim of CURE Soft is to provide clinicians with real-time information about a patient’s respiratory mechanics based on current ventilator settings and breath-to-breath data in a simple graphical format. The information is translated into time-series and pressure dependent graphs to help clinicians to select optimal ventilator settings. CURE Soft focuses on using model-identified, patient-specific respiratory mechanics parameters to help select a PEEP at minimal respiratory elastance [2, 18]. Figure 1 shows a schematic of how CURE Soft is implemented in the intensive care unit (ICU) to support clinical decision making.

### Time-varying elastance single compartment model

CURE Soft calculates respiratory mechanics parameters using an extended single compartment lung model which captures time-varying respiratory elastance model [2, 19]. The model is described by Equations (1) and (2). This model is extended from the single compartment model and uses the measured airway pressure and flow of the ventilator to calculate the respiratory elastance and resistance for each breathing cycle. The model description can be found in Table 1.
12

*V(t)* is calculated by integrating the flow, *Q(t). E*_*rs*_ and *R*_*rs*_ can be determined using multiple linear regression [19, 20]. Using the *R*_*rs*_ value obtained from Equation (1), the time-varying respiratory elastance (*E*_*drs*_) within the inspiration of each breathing cycle is calculated using Equation (2) [19]. Each breathing cycle value for *E*_*drs*_ is then normalised to the total inspiration time, and the area under the *E*_*drs*_ curve (*AUCE*_*drs*_) is used to calculate *Stiffness* (The term *Stiffness* is used in the CURE Soft for simpler understanding of non-engineering audience). This normalisation allows comparison across different respiratory rates within a given ventilation mode, or across different ventilation modes where respiratory rate may vary.

**Table 1:** **Model descriptions**

Symbols	Description	Units
*P*_*aw*_	Airway pressure in a breathing cycle	cmH_2_O
*t*	Time	s
*E*_*rs*_	Respiratory system elastance	cmH_2_O/l
*V*	Air volume	l
*R*_*rs*_	Respiratory airway resistance	cmH_2_Os/l
*Q*	Airway flow	l/s
*P0*	Offset pressure	cmH_2_O

### Data acquisition, management and processing

Airway pressure and flow data is obtained from the Puritan Bennett 840 ventilator through the ventilator’s waveforms output function. The RS-232 serial port on the rear of the ventilator display is connected to a laptop running CURE Soft through an external USB adapter and a USB cable. The serial port was set up in JAVA with a baud rate of 38400, to match the ventilator output. A callback function is used to read all the available data and stores it in text format. In this data, two characters “BS” and “BE” can be found. These characters signify when breathing starts and ends respectively. “BS” appears when the ventilator starts to provide breathing support and “BE” appears at the end of the breathing cycle. Following the “BE”, the starting point of a subsequent breath “BS”, appears right after the previous “BE”, and this cycles continue until the ventilator is disconnected or stopped providing support.

Patient-specific *E*_*rs*_ is calculated from the single compartment lung model using only the inspiratory portion of breathing [2, 19]. Each breathing cycle is separated into inspiration and expiratory sections. The occurrence of the “BS” character signifies the beginning of inspiration, and the next occurrence of these characters is the end of expiration. The end of inspiration is determined by searching for the first point where air flow changes from being positive to negative. In addition, breathing cycle filtering is performed, by quarantining ‘abnormal’ breathing cycles. These breathing cycles are too short, too long, non-physiological plausible or the breathing cycle data that are corrupted during data acquisition process. In particular, the filtering criteria are as follows:

-  A stream of data that does not contain both BS and BE
-  A sudden pressure difference with more than ± 10 cmH_2_O compared to previous point
-  A sudden flow difference with more than ± 10 cmH_2_O/l compared to previous point
-  A ‘breathing cycle’ with less than 5 data points is not processed

### Software architecture

The architecture of the CURE Soft is designed according to the classical three-layer structure. The lowest layer is responsible for the persistent data storage and for the management of input ports. The input port management is isolated from the other parts of the system by dedicated objects in order to provide the opportunity of easy replacement of the used ventilator device. The middle layer implements the data processing and management functions in a modular way. Thus, the current set of functions and model calculations can be easily extended. The highest layer of the system provides the graphical user interface (GUI).

### Graphic user interface and main user function

CURE Soft can be used in two modes of operation:

1. *Online*: This mode acquires data from the ventilator in real-time and calculates the respiratory mechanic parameters in real-time to aid clinical decision making. This mode also stores the real-time data and relevant input to a text format.
2. *Offline*: This mode allows access to the data stored during online use for simulation. It provides the opportunity for user education purposes and auditing or reviewing patient care and progression.

The general procedure to execute CURE Soft is shown in Table 2.

**Figure 2:**
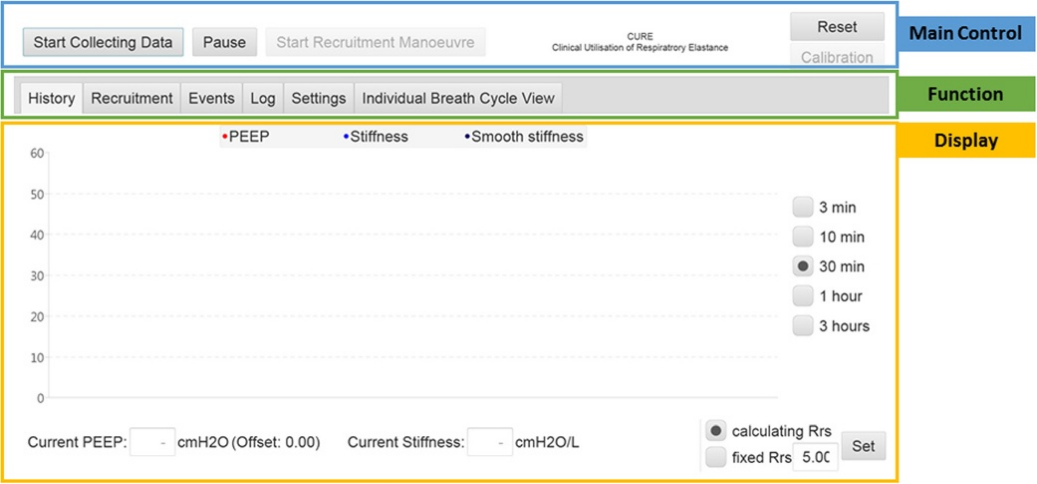
**CURE GUI upon execution.** 1) Main Control Panel, 2) Function Panel, 3) Display Panel. The font size of the GUI has been modified in this figure for display.

Upon execution, the CURE Soft graphical user interface (GUI) is as shown in Figure 2. The CURE Soft GUI is separated into three sections: 1) Main Control, 2) Function and 3) Display. The buttons in Main Control are used for initiation and termination of the CURE Soft.

**Table 2:** **CURE Soft application procedure**

	Online mode		Offline mode
**Ventilator settings**			
1.	Check ventilator output, network communication, output waveform is selected with baud rate 38400.		-
2.	Connect an RS232 serial port from the rear of the ventilator to the computing platform with CURE Soft.		-
**Computer settings**			
1.	Execute CURE Jar.	1.	Execute CURE jar.
2.	In settings tab, select serial port	2.	In settings tab, select captured file.
3.	Select the serial port connected to the RS232.	3.	Select the designated captured file (*.txt).
4.	Click ‘start collecting data’.	4.	Click ‘start collecting data’.

The Functions Panel is where the different CURE Soft functions are located. There are 6 main function tabs in the CURE Soft GUI with their functions summarised below:

1. *History*: Display the history of calculated *Stiffness*, PEEP and average *Stiffness*.
2. *Recruitment*: This tab is used during a recruitment manoeuvre (RM) or PEEP titration procedure. *Stiffness* at each PEEP is calculated and evaluated. The PEEP before minimal *Stiffness* or *Stiffness* occurs is selected as model-selected PEEP.
3. *Events*: This tab allows additional clinical events and procedures to be recorded.
4. *Log*: Log file for software evaluation purposes.
5. *Settings*: Settings for CURE Soft application. Settings include aspects like: 1) *Online* or *Offline* mode; 2) Where to store specific data files.
6. *Individual Breath Cycle View*: Allows every calculated parameter to be viewed at every evaluated breathing cycle.

Finally, the display section is used to present the results calculated from the models and data. A step-by-step guide in video format is included in the electronic supplemental file to provide offline training for the use of the CURE Soft application (See Additional files 1, 2, 3, 4, 5, 6 and 7).

### Software testing

Unit and integration tests of CURE Soft were regularly executed during the development of the application. Occasionally, previously captured data sets were used for these test cases. At the end of the development cycle, the functions of CURE Soft application were validated in two phases. First, CURE Soft was tested using a mechanical lung (Michigan Instruments Dual Adult Test Lung) connected the Puritan Bennett 840 (PB840) ventilator. To validate the CURE Soft application in clinical settings, a clinical trial is being carried out to investigate the potential of using respiratory mechanics to optimise PEEP in MV in Christchurch Hospital, New Zealand (Australian New Zealand Trial Registry Number: ACTRN12613001006730). Ethics approval for this study was granted by the New Zealand Southern Regional Ethics Committee (Reference number: 13/STH/84). Written informed consent was obtained from the patient for the publication of this report.

In this manuscript, the data from a patient who was mechanically ventilated invasively due to respiratory failure is included for software validation testing. This patient was intubated and ventilated using a Puritan Bennett 840 ventilator, under synchronous intermittent mandatory ventilation (SIMV) mode, volume control with tidal volume (6 ~ 8 ml/kg). The patient was later sedated and paralysed to undergo a RM for PEEP titration. These sections of data are used to demonstrate the program and its functionality.

It is important to note that while a PB840 ventilator is used in the development and testing, the software is fully general. Using the architecture as described above, implementation of the CURE Soft in other ventilators only requires changing the data acquisition and processing to allows access to pressure and flow data of that specific ventilator.

## Results and discussion

### CURE Soft trial and general observations

**Figure 3:**
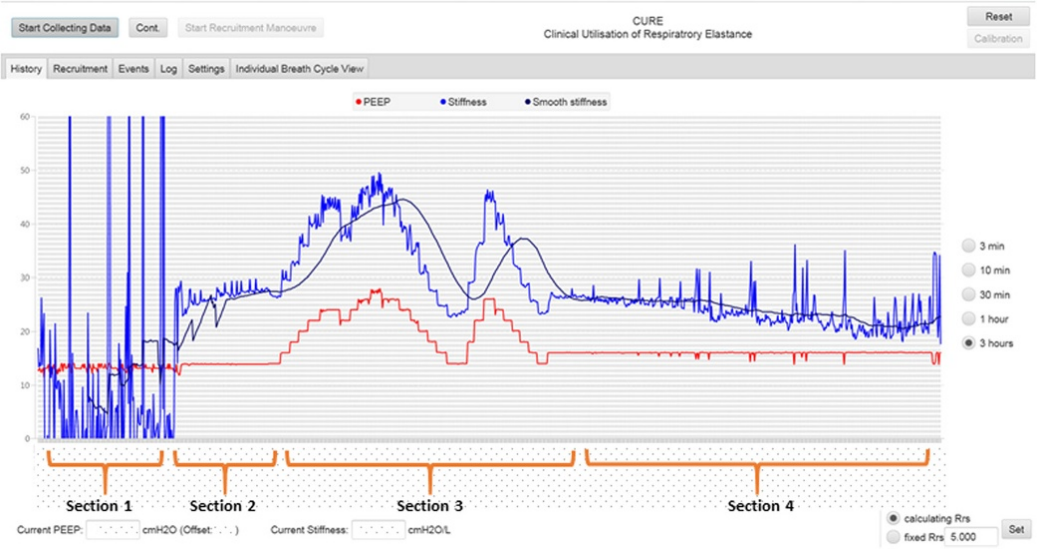
**Implementation of CURE Soft in the patients included in the software testing trial.** The software testing can be divided into 4 sections. Section 1: When patient exhibits spontaneous breathing effort. Section 2: Patient is paralysed to prevent spontaneous breathing efforts. Section 3: Patient undergoes a recruitment manoeuvre and a PEEP titration manoeuvre. Section 4: Ventilator PEEP adjusted and Patient slowly regains spontaneous breathing efforts.

At the start of the clinical trial, CURE Soft is initiated to calculate respiratory system *stiffness*. Figure 3 shows the respiratory mechanics calculated for the patient over different periods of time. The blue line shows the calculated respiratory system *AUCE*_*drs*_, or ‘*stiffness*’. The black line indicates is the smooth *stiffness* averaged over the last 60 breathing cycles. The red line is the PEEP detected from the ventilator airway pressure. The result shown in Figure 3 is divided into 4 sections.

-  
*Section 1:* Respiratory mechanics were calculated at the start of the clinical trial and patient was spontaneously breathing on top of ventilator support.
-  
*Section 2*: Patient is paralysed to prevent spontaneous breathing efforts.
-  
*Section 3*: Once patient is paralysed, a protocolised RM is performed. The PEEP is increased in steps of 2 cmH_2_O until peak airway pressure reached 55 cmH_2_O, then decrease back to initial PEEP. A second RM is performed to titrate PEEP and validate the initial findings during the first RM.
-  
*Section 4*: Ventilator PEEP is selected based on the CURE Soft recommendation and clinician consensus. Patient slowly regain spontaneous breathing efforts.

In Section 1, the patient respiratory *stiffness* fluctuates and is sometimes reduced to non-physiological value of less than zero. These non-physiological values occurred due to the patient’s added spontaneous breathing effort [21, 22]. Patients who are spontaneously breathing are more variable, and thus, each breathing cycle is altered by the variable pressure generated by the diaphragm and/or intercostal muscles. These variable efforts significantly altered the airway pressure and flow data, affecting the respiratory mechanics and parameter identification process. The ability of CURE Soft to calculate an effective, net patient-specific respiratory mechanics values is limited when the patient is breathing spontaneously due to incorrect modelling approach [23]. When the patient is spontaneously breathing, there is a need of additional measuring tools to isolate patient-specific breathing effort for patient-specific respiratory mechanics calculation [24].

In Section 2, the patient was given muscle relaxants for paralysis. Once the patient was paralysed, the *stiffness* fluctuation in Section 1 ceased almost immediately. From this point, the true patient-specific respiratory mechanics are no longer altered by the patient’s inspiratory effort, and the mechanical parameter values are reliable.

**Figure 4:**
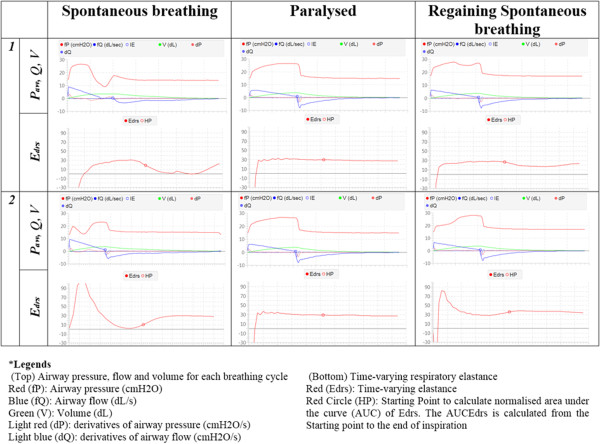
**6 individual breathing cycles monitoring in the CURE Soft GUI.** Left column: Breathing cycles severely altered due to spontaneous breathing efforts, Middle Column: Breathing cycles after muscle paralysis and, Right Column: Breathing cycles when the patient starts to regain spontaneous breathing efforts.

Figure 4 shows the airway pressure, flow, volume and calculated time-varying elastance (*E*_*drs*_) curve for several breathing cycles before and after paralysis. It is important to note that, if the calculated respiratory mechanics are used to guide ventilation, the respiratory mechanics model must provide unique solutions without a wide range of variation. Thus, in this study, since the model was not able to account for patient variability during Section 1 of the trial, patients was given muscle relaxants to reduce this variability.

**Figure 5:**
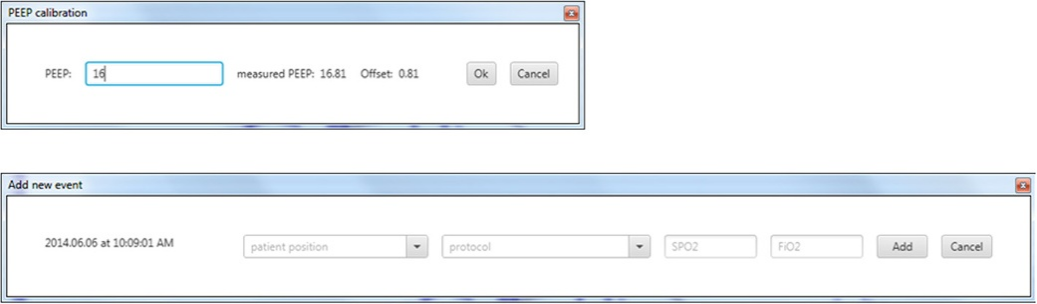
**CURE GUI pop-up dialog.** Top: PEEP calibration and, Bottom: Events Log.

Once the patient was paralysed and the respiratory mechanics could reliably be calculated, a step-wise PEEP increase RM was performed to evaluate the respiratory *stiffness* at each PEEP level [2, 19]. A second RM was also performed for validation and PEEP titration. At the start of the first RM, the ‘Start Recruitment Manoeuvre’ button is clicked, and the CURE Soft user is required to calibrate the ventilator displayed PEEP with actual measured PEEP. After the PEEP is calibrated, an event log is also available to record the patient’s position, protocol, pulse oximetry oxygen saturation (SpO_2_) and fraction of inspired oxygen (FiO_2_). The calibration dialog and event logs are shown in Figure 5.

**Figure 6:**
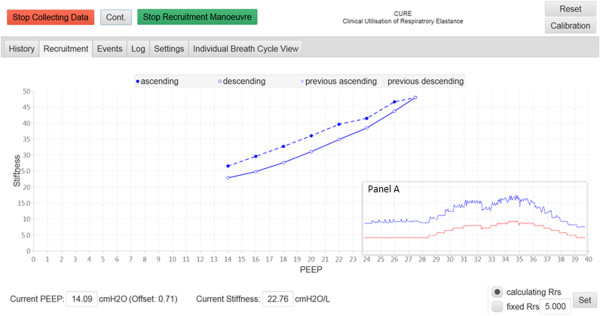
**Stiffness (Respiratory Elastance) vs PEEP during the first step-wise PEEP recruitment manoeuvre.** The dashed lines are during ascending PEEP, and solid lines are during descending PEEP. Panel A shows the *Stiffness*-time and PEEP-time plot in 30 minutes time scale.

**Figure 7:**
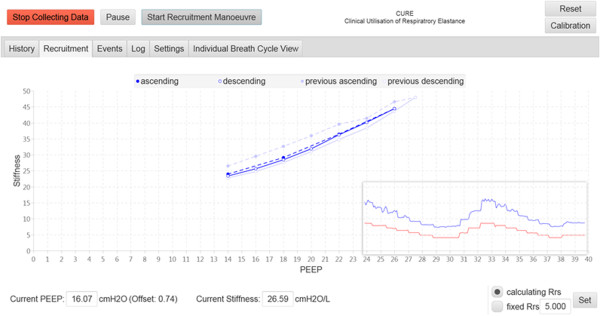
**A second recruitment manoeuvre right after the first recruitment manoeuvre.**

After the events are recorded, the History Tab display is switched to the Recruitment Tab display. This tab displays the respiratory system *stiffness* on the y-axis and with PEEP on x-axis as shown in Figures 6 and 7. The plots in Figures 6 and 7 correspond to the respiratory mechanics in *Section 3*, Figure 3.

During the first RM as shown in Figure 6, the PEEP was increased step-wise from initial value of 14 cmH_2_O until PEEP level of 27.5 cmH_2_O. The PEEP is then decreased step-wise to the initial PEEP setting. The purpose of the RM is to recruit collapsed lung. As the PEEP is decreased back to the initial PEEP, there is a noticeable hysteresis between *stiffness* during PEEP increase, and *stiffness* during PEEP decrease. This hysteresis indicates additional alveoli recruitment. As PEEP is decreased from higher PEEP, the threshold closing pressures of these newly recruited alveoli are lower than their threshold opening pressure, and thus these alveoli remain opened.

**Figure 8:**
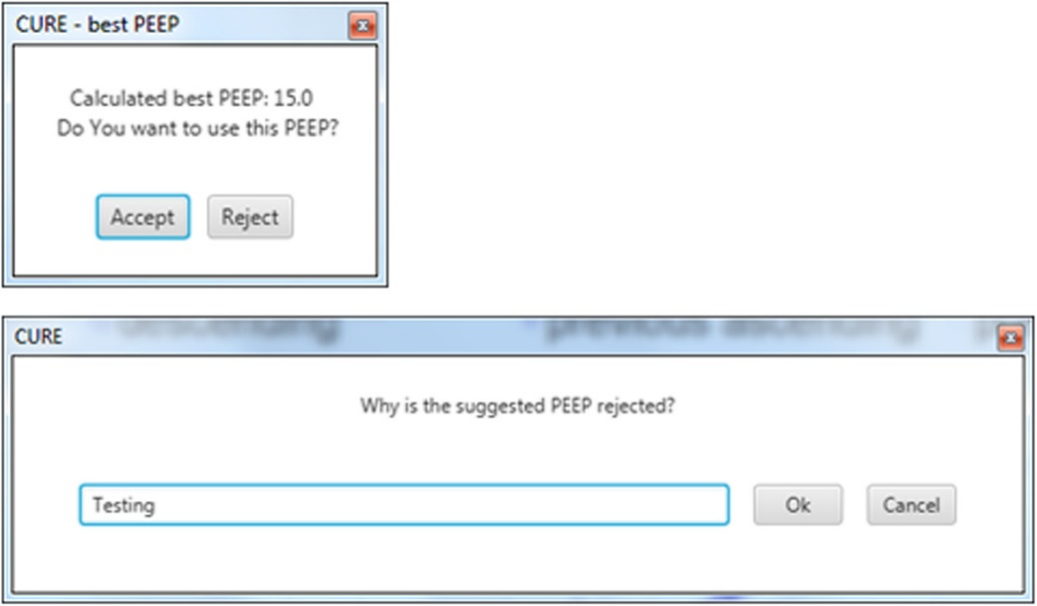
**PEEP is recommended to the CURE Soft user once ‘Stop Recruitment Manoeuvre’ button is clicked.** It is at the user discretion to follow the suggestion.

At the end of the RM, the ‘Stop Recruitment Manoeuvre’ button in Figure 6 is clicked. This action will trigger the PEEP selection function, where a PEEP will be suggested by the CURE Soft based on inflection elastance (in this case, a PEEP of 15 cmH_2_O is recommended) [2]. This CURE Soft suggested PEEP occurs right before the minimal elastance (*stiffness*) PEEP, which aim to maintain time-dependent alveoli recruitment and a balance of recruitment and overdistension [19]. The user is then given an option to accept the suggested PEEP or not, as shown in Figure 8. CURE Soft GUI aims to provide decision support in PEEP selection. It is important that the final clinical decision is confirmed in CURE Soft by the attending clinicians. Following the first RM, a second RM was performed to titrate PEEP again using the ‘Start Recruitment Manoeuvre’ button. The result of the second RM is as shown in Figure 7. The first or previous RM stiffness-PEEP plot is recorded in the background of the display for comparison with the newer RM. During the second RM and compared to the previous RM, it is obvious that the *stiffness*-PEEP curve during increasing and decreasing PEEP were effectively overlaid. This result suggested that the first RM was able to ‘fully’ recruit the recruitable collapsed alveoli and there is relatively little or no alveoli collapse after the first RM. At the end of the RM, the *Stiffness*-PEEP curve is again evaluated and a PEEP of 15 cmH_2_O is recommended as optimal ventilator PEEP. However, as this was a trial for software testing, and attending clinicians decided to select a PEEP of 16 cmH_2_O.

After the PEEP was set at 16 cmH_2_O, the RMs were stopped, but the patient’s respiratory mechanics, together with airway pressure and flow profiles, were continuously recorded until the end of *Section 4* as shown in Figure 3. From *Section 3* to *Section 4*, it was found that the respiratory *stiffness* fluctuates at the end of the trial. This result suggested that the patient started to regain spontaneous breathing effort. This fluctuation of respiratory *stiffness* corresponds to the ‘entrainment’ of breathing on MV as shown in Figure 4 (Right Column) where the shape of the airway pressure is altered by the patients breathing effort [21, 22].

### Application of CURE Soft in respiratory mechanics monitoring

CURE Soft identifies patient-specific respiratory mechanics (elastance or *stiffness*) in real-time from ventilator data. Calculations of respiratory mechanics were previously available by performing specific intensive clinical protocols [25]. The existing methods of respiratory mechanics monitoring are invasive and/or interrupt care, and thus cannot be performed continuously. In particular, most ventilator continuous monitoring methods use two point respiratory mechanics estimation, which can only be performed in one specific set ventilation mode using an additional end of inspiratory pause (EIP) [26], which can be automated by some ventilators (For example: the Engstrom Carestation and Puritan Bennett 980). Equally, this method heavily relies on the duration of the EIP [26, 27] and provides only a local estimation at that point and pressure of the breath, which may not match what is obtained via a model-based approach that requires no EIP. Thus, in comparison, CURE Soft uses a model-based approach using readily available airway pressure and flow profile, adds no additional burden to patients, and provides more information than was previously unavailable. Clinically, the CURE Soft system allows continuous tracking of patients’ respiratory mechanics across time. Information about changing patient condition, and response to treatment over time is very useful, as it can be used to evaluate the efficacy of previous treatments and regularly audit patient care and progression.

CURE Soft also provides real-time feedback on the effect of a RM, and allows clinical staff to determine a patients’ lung recruitability, and suggest an optimal patient- and time- specific PEEP level. Finally, as shown here, monitoring respiratory mechanics continuously can also provide information about patient-ventilator interactions and thus, potentially provide useful insight to anaesthesia, analgesia and overall patient ventilator management that was not necessarily previously available to clinicians.

### Limitations and future works

CURE Soft was able to calculate patient-specific respiratory mechanics for every breath using the airway pressure and flow information from the ventilator. However, as shown in the results, the single compartment model used by CURE Soft is less reliable if the patient is breathing spontaneously, although this issue can be managed [28]. In particular, patients with spontaneous breathing, create a pressure or flow drop during a breathing cycle, as shown in Figure 4 (Left and Right Column). This pressure drop alters the shape of the airway pressure or flow curve of that breathing cycle, and thus, the calculated respiratory mechanics are over or under estimated. While this limitation can be overcome with the use of muscle relaxants, it was reported that the use of muscle relaxants are generally not clinically feasible and a more advanced models are required [28]. Thus, the application of CURE Soft is currently limited to patients who are synchronised with the ventilation support without ‘entrainment’ [22]. However, the addition of the advanced model presents no hurdle or significant change to the existing GUI.

In this study, the CURE Soft was tested in human trials using a SIMV volume control mode with ramp flow profile. This flow profile is similar to the flow profile generated during pressure control mode. Thus, the CURE Soft has similar ability to capture the respiratory mechanics during pressure control modes. This ability was tested under different controlled ventilation using the mechanical lung (see Additional file 1). However, the application of CURE Soft during pressure control mode warrants further investigation in human subjects.

There is also a need to further study of the effect of optimising PEEP on patient recovery. CURE Soft selects a PEEP slightly higher than the minimal elastance PEEP as seen in Figure 7. This higher PEEP is selected to maintain alveoli recruitment aside from maintaining a balance in recruitment and overdistension [19]. A recent study by Pintado et al. found that selecting PEEP based on a metric similar to minimal respiratory elastance may potentially improve patient recovery [29]. Thus, the application of CURE Soft, is a system necessary to test this hypothesis at scale without clinically intensive and thus, infrequent interventions.

## Conclusions

CURE Soft enables the real-time calculation of model-based respiratory mechanics for patients receiving mechanical ventilation. It is also capable of providing a unique training and auditing tool for clinical users. Initial results from the clinical trials, showed that the system is able to provide detailed, previously unavailable information on patient-specific respiratory mechanics and response to therapy in real-time. The additional insight available to clinicians provides the potential for improved decision-making, and thus improved patient care and outcomes.

## Availability and requirements

**Project name:** Clinical Utilisation of Respiratory Elastance Software (CURE Soft)

**Project home page:** not available

**Operating system(s):** Windows, Linux

**Programming language:** JAVA 7

**Other requirements:** JAVA JRE7, Mechanical Ventilator (CURE Soft was tested on Puritan Bennett 840 and Puritan Bennett 980)

**Any restrictions to use by non-academics:** no restrictions

## Authors’ information

AS and BB works at Department of Control Engineering and Information, Budapest University of Technology and Economics. YSC, DR, AB, DG, SC, VM, CP and JGC are from the Centre for BioEngineering, University of Canterbury. GMS works in the Department of Intensive care in Christchurch Hospital. TD works at the GIGA-Cardiovascular Sciences, University of Liege.

## Electronic supplementary material

Additional file 1: **Electronic Supplemental File.** (DOCX 9 MB)

Additional file 2: **Video 1.** (AVI 16 MB)

Additional file 3: **Video 2.** (AVI 9 MB)

Additional file 4: **Video 3.** (AVI 5 MB)

Additional file 5: **Video 4.** (AVI 13 MB)

Additional file 6: **Video 5.** (AVI 1 MB)

Additional file 7: **CURE_v1.0.11-experimental.** (ZIP 4 MB)

## Abbreviations

ARDS: Acute respiratory distress syndrome
CURE: Clinical utilisation of respiratory elastance
USB: Universal serial bus
Soft: Software
GUI: Graphic user interface
*E*_*drs*_: Time-varying respiratory elastance
*E*_*rs*_: Respiratory system elastance
*R*_*rs*_: Respiratory system resistance
*P*_*aw*_: Airway pressure
*Q*: Airway flow
*V*: Air volume
*P0*: Offset pressure or positive end-expiratory pressure
PB840: Puritan Bennett 840 ventilator
MV: Mechanical ventilation
SpO_2_: Pulse oximetry oxygen saturation
FiO_2_: Fraction of inspired oxygen
PEEP: Positive end-expiratory pressure
RM: Recruitment manoeuvre
ICU: Intensive care unit.
