# Supplementary material for: The Clinical Utilisation of Respiratory Elastance Software (CURE Soft): a bedside software for real-time respiratory mechanics monitoring and mechanical ventilation management
Source: Biomed Eng Online. 2014 Sep 30;13:140. doi: 10.1186/1475-925X-13-140 (PMC4192763; doi:10.1186/1475-925X-13-140)
Supplement: Supplementary file 1 — Additional file 1: Electronic Supplemental File. (DOCX 9 MB) [file 12938_2014_875_MOESM1_ESM.docx]

**Electronic Supplemental File**

There are 5 sections in electronic supplemental file.

**Section A: Using CURE.jar**: CURE.jar installation and using it

**Section B: Video Tutorials**: Step-by-step video tutorial for CURE.jar

**Section C: CURE Soft Graphic User Interface**: General description of CURE.jar

**Section D: CURE Soft Model Testing with Mechanical Lung**: CURE model testing in different ventilation mode using a mechanical lung

**Section E: Phase Lag due to Moving Average**: CURE Soft moving average selection and phase lag description

**A. Using CURE.jar**

1. Download the compressed electronic supplemental file.
2. Extract the contents and run CURE.jar.
3. Patient example used in the video tutorial (Patient8.txt) is included in the compressed folder.
4. Java SE Runtime Environment 7 is required to use CURE.jar.
5. Follow the video tutorials in Section B.
6. The general description of all CURE Soft GUI function and display are detailed in Section C.

**B. Video Tutorials**

There are a total of 5 video files submitted along with the manuscript and software. These video files will provide a step-by-step guide for user to run the CURE Soft in Offline mode on a patient sample data. These 5 videos are in arranged in chronological order, but are separated to describe different scenarios. Each video file has a length of 5 to10 minutes.

The videos are uploaded separately as electronic supplemental files. The videos are also available online in dropbox with the download links shown below.

**Video 1:** <https://www.dropbox.com/s/disg42k5gyyawop/Ver%202%20-%201.avi>

**Video 2:** <https://www.dropbox.com/s/g7jozyscdpm76y5/Ver%202%20-%202.avi>

**Video 3:** <https://www.dropbox.com/s/ljtg983lrcdfli1/Ver%202%20-%203.avi>

**Video 4:** <https://www.dropbox.com/s/5r8hyt8h30rzxgl/Ver%202%20-%204.avi>

**Video 5:** <https://www.dropbox.com/s/g6d71sxuknnm1d2/Ver%202%20-%205.avi>

The videos are video only tutorial (no sound). The videos were recorded using Bandicam 2.0.0.638 (Copyright © 2009-2014 Bandisoft.com, unregistered with 10 minutes video with water mark). The recorded videos had playing speed increased by approximately 33% compared to the real-time offline simulation. The speed is increased to reduce the total time of the video while maintaining the important messages. It is recommended that the video player scrollbar is used to move to the event as listed in the tables below.

The following tables summaries the important event shown the video tutorial.

**1. Video File 1:**

This video presents the initiation of the CURE Soft.

| **Time** | **Tab** | **Activity Description** |
| --- | --- | --- |
| 0:10 | Settings | Setting up Subfolders to load offline file. |
| 0:26 | History | Click Start Collecting Data Button. |
| 0:37 | Events | Record Events: Patient position, Description of Events, SpO2 and FiO2. |
| 1:05 | Log | Logs for CURE Soft. |
| 1:28 | History | Check *Stiffness* (Blue Line) and PEEP (Red Line). |
| 1:49 | Individual | Click Start listening to monitor each breathing cycle’s airway pressure, flow, and other calculated parameters. |
| 2:27 | History | Check *Stiffness* and PEEP (Occurrence of unstable non-plausible *Stiffness*). |
| 2:35 | History | After 60 breathing cycles occurred, a smooth *Stiffness* (Average of the 60 breaths *Stiffness*) is displayed as Black Line.  *The CURE Soft smooth *Stiffness* can be modelled at any number of breathing cycles depending on the user preference. In this training video, it is set at 60. This moving average thus result in a phase lag if sudden respiratory *Stiffness* is changed suddenly. The phase lag comparison is shown in the end of this document |
| 2:50 | Individual | Each breathing cycle has altered and variable airway pressure due to spontaneous breathing efforts causing the *Stiffness* to be variable. |
| 5:45 | Individual | Patient was given muscle relaxants. The airway pressure curve became ‘normal’. |
| 6:40 | Event | Record the event of muscle paralysis. |
| 7:15 | Calibration | Once patient is paralysed, PEEP calibration is performed. |
| 8:04 | History | Pause Button is used to stop the simulation and allow time for training purposes. |

**2. Video File 2:**

This video presents the first recruitment manoeuvre performed to the patient to recruit the lung.

| **Time** | **Tab** | **Activity Descriptions** |
| --- | --- | --- |
| 0:05 | History | Cont. Button is used to continue with the previous simulation. |
| 0:11 | History | Once patient is paralysed, a recruitment manoeuvre can be performed.  Click Start Recruitment Manoeuvre Button.  Perform PEEP Calibration (14 cmH_2_O).  Event Tab is triggered to record the event. |
| 0:44 | Recruitment | *Stiffness* vs PEEP is plotted on the Display panel. |
| 2:12 | Recruitment | CURE Soft detects the PEEP change on the ventilator.  A yellow signal is displayed during the PEEP change.  Once *Stiffness* vs PEEP plot for the next PEEP level is displayed, the signal turns green.  The cycles then repeats for every PEEP change.  The yellow and green signal indicates the buffer time to allow a short time period for the stiffness to be stabilised. |
| 4:31 | Recruitment | PEEP is decreased by one step and increase back to same value. |
| 6:04 | Recruitment | PEEP reaches maximum allowable airway pressure and begin PEEP decremental. |
| 8.47 | Recruitment | PEEP is decreased to initial value.  Click stop recruitment manoeuvre. Once the recruitment manoeuvre is stopped, the decreasing portion of the *Stiffness* vs PEEP curve is evaluated and the CURE Soft recommends a PEEP value.  Suggested PEEP is rejected due to ‘Testing’. |
| 8:55 | Recruitment | Pause Button is used to stop the simulation and allow time for training purposes. |

**3. Video File 3:**

This video presents the 2 recruitment manoeuvre performed to the patient to validate the first recruitment manoeuvre as well as to perform PEEP titration.

| **Time** | **Tab** | **Activity Descriptions** |
| --- | --- | --- |
| 0:01 | Recruitment | Cont. Button is used to continue with the previous simulation. |
| 0:04 | Recruitment | Click Start the Recruitment Manoeuvre Button.  Perform PEEP Calibration (14 cmH_2_O).  Event Tab is triggered to record the event. |
| 0:18 | Recruitment | The previous Recruitment Manoeuvre *Stiffness* vs PEEP graph is displayed at the background for comparison to the newer Recruitment Manoeuvre |
| 0:24 | Recruitment | *Stiffness* vs PEEP is plotted on the display panel. |
| 1:35 | Recruitment | Upon reaching maximum PEEP, PEEP is decreased step-wise to initial PEEP. |
| 1:38 | Recruitment | PEEP begins to decrease to initial PEEP. |
| 3:23 | Recruitment | PEEP is decreased to initial value.  Click stop recruitment manoeuvre. Once the recruitment manoeuvre is stopped, the decreasing portion of the *Stiffness* vs PEEP curve is evaluated and the CURE Soft recommends a PEEP value.  Suggested PEEP (15 cmH_2_O) is rejected due to ‘Testing’. |
| 3:40 | History | At this stage Patient ventilator PEEP is increase to 16 cmH_2_O at clinician’s discretion. |
| 4:00 | Events | Record Events “CURE Soft Test Complete”. |
| 5:03 | History | Pause Button is used to stop the simulation and allow time for training purposes. |

**4. Video File 4:**

This video presents the CURE Soft *Stiffness* estimation using fixed airway resistance and compared with variable airway resistance. The simulation is continued until the patient slowly regains muscle activity.

| **Time** | **Tab** | **Activity Descriptions** |
| --- | --- | --- |
| 0:04 | History | Cont. Button is used to continue with the previous simulation. |
| 0:20 | Individual | Calculating Rrs is switched to Fixed Rrs. Click Set  This function enables Rrs to be constant and allows Ers and *Stiffness* comparison |
| 1:04 | History | Fixed Rrs is switched to Calculating Rrs. Click Set |
| 8:12 | History | As time progresses, the *Stiffness* regains variability, indicating muscle relaxants wearing off. |
| 9:02 | Events | Add events indicating patients regains spontaneous breathing effort. |
| 10:00 | History | Click Pause |

**5. Video File 5:**

This video presents the final section for the simulation.

| **Time** | **Tab** | **Activity Descriptions** |
| --- | --- | --- |
| 0:03 | History | Cont. Button is used to continue with the previous simulation. |
| 0:30 | History | Click Stop Collecting Data Button |
| 0:53 | History | Click Reset to clear all data |

**C. CURE Soft Graphic User Interface**

The following shows the CURE Soft GUI general description.

C1. Main GUI and History Tab


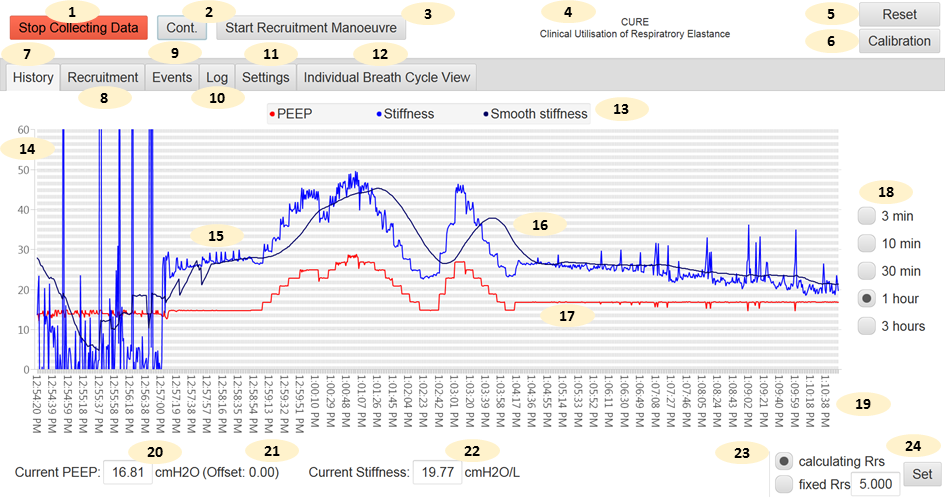


| **No.** | **Function Description** |
| --- | --- |
| 1 | Start and Stop button for data collection. |
| 2 | Pause and Continue button if used in offline mode. |
| 3 | Start and Stop button for recruitment manoeuvre and PEEP titration button |
| 4 | Software name |
| 5 | Reset button |
| 6 | Calibration button to calibrate measured PEEP to ventilator set PEEP |
| 7 | History function tab |
| 8 | Recruitment function tab |
| 9 | Events function tab |
| 10 | Log function tab |
| 11 | Setting function tab |
| 12 | Individual breath cycle display tab |
| 13 | Graph legends |
| 14 | Y-axis (PEEP in cmH_2_O and *Stiffness* in cmH_2_O/L) |
| 15 | *Stiffness* (blue line) |
| 16 | Smooth *Stiffness* (black line) is calculated over a moving window of 60 breathing cycles |
| 17 | Positive end expiratory pressure (PEEP) (red line) |
| **No.** | **Function Description** |
| 18 | Time scale |
| 19 | X-axis (Time in AM/PM) |
| 20 | Current PEEP measure from Airway pressure (Calibrated with ventilator set PEEP) |
| 21 | PEEP offset (Difference between airway pressure measured PEEP and ventilator set PEEP) |
| 22 | Current *Stiffness* (cmH_2_O) |
| 23 | Selecting a model-based estimated airway resistance or fixed airway resistance (*R_rs_* in cmH2Os/l) |
| 24 | Set airway resistance configuration |

C2. Recruitment Tab


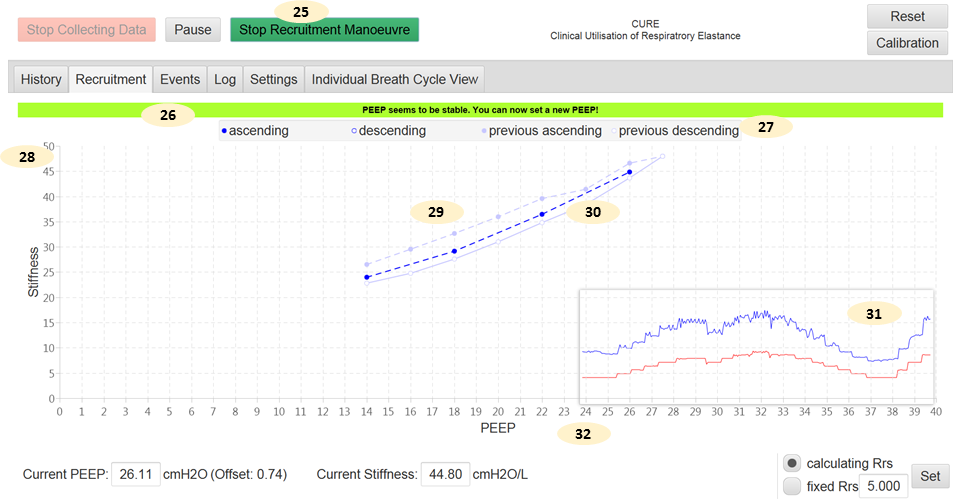


| **No.** | **Description** |
| --- | --- |
| 25 | Start and stop recruitment manoeuvre function |
| 26 | PEEP change indicator (Allows the *Stiffness* to be plotted) |
| 27 | Figure legend |
| 28 | Y-axis (*Stiffness* in cmH_2_O) |
| 29 | Previous *Stiffness*-PEEP plot |
| 30 | Current *Stiffness*-PEEP plot |
| 31 | *Stiffness*-time and PEEP-time plot in 30 minutes time scale (For reference on how much time have elapsed) |
| 32 | X-axis (PEEP in cmH_2_O) |

C3. Events Tab


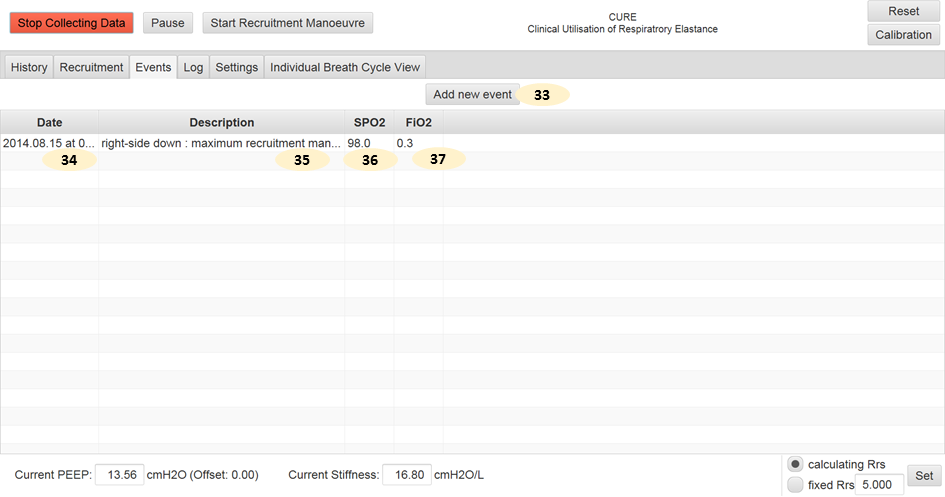


C4. Log Tab


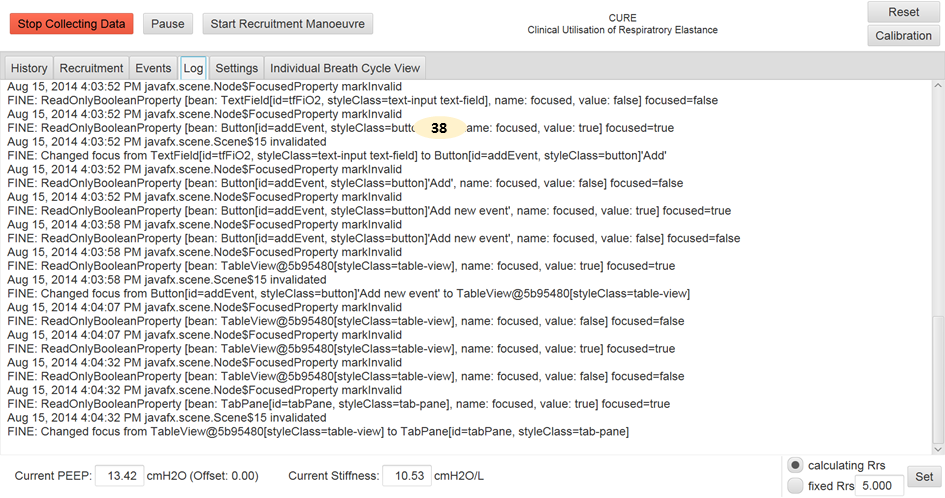


| **No.** | **Description** |
| --- | --- |
| 33 | Add new event function |
| 34 | Date and time of the event |
| 35 | Description of the event (Patients position and description) |
| 36 | Patient oxygen saturation measured by pulse oximetry (SPO_2_ in percentage, eg. 97) |
| 37 | Ventilator set fraction of inspired oxygen (FiO_2_ in fraction, eg. 0.4) |
| 38 | CURE Soft program log for feedback and debugging |

C5. Settings Tab


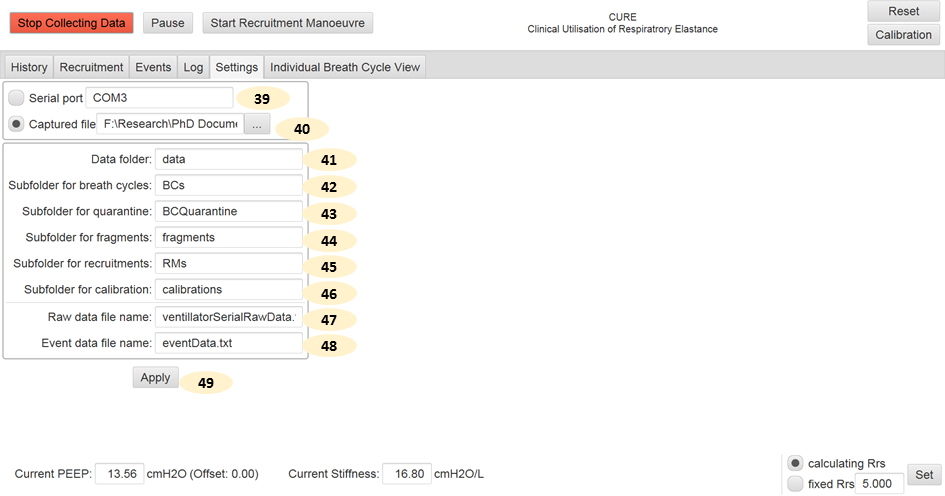


| **No.** | **Description** |
| --- | --- |
| 39 | CURE Soft online mode and select the serial port connecting to the ventilator |
| 40 | CURE Soft offline mode and select the recorded data for review and auditing purposes |
| 41 | Name folder for data storage |
| 42 | Name subfolder for breathing cycle storage |
| 43 | Name subfolder for quarantine breathing cycle storage |
| 44 | Name subfolder for data fragments storage (only available if data processing fail and used only for debugging) |
| 45 | Name subfolder for recruitment manoeuvre storage (only available if any recruitment manoeuvre is performed, and the function is used) |
| 46 | Name subfolder for storing breathing cycles used in calibration (only available if any PEEP calibration is performed) |
| 47 | Name of the raw ventilator data file |
| 48 | Name of the event data file |
| 49 | Apply the settings |

C6. Individual Breath Cycle View Tab


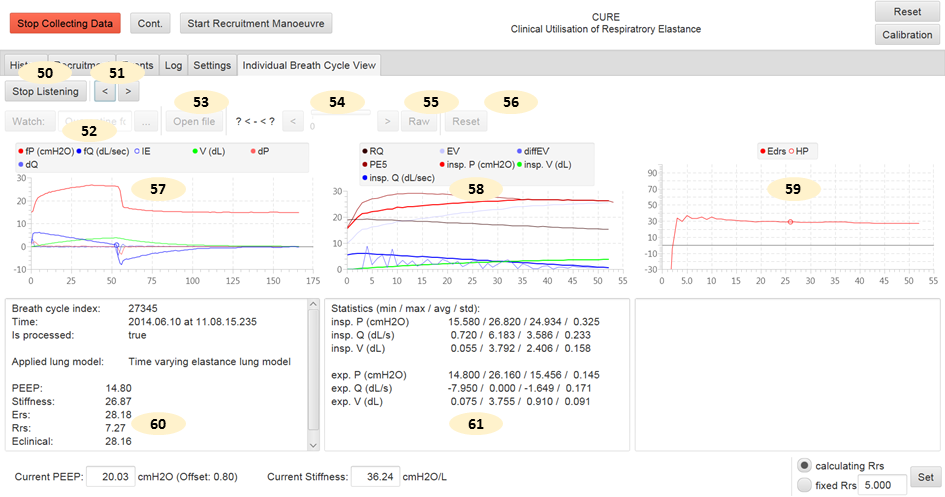


| **No.** | **Description** |
| --- | --- |
| 50 | Listening function (Capturing and viewing each individual breathing cycle) |
| 51 | Select breathing cycle to view (Only available during offline, CURE soft is paused and when there are stored individual breathing cycle) |
| 52 | Watch folder (designated to view breathing cycles for improved data processing) |
| 53 | Open a specific breathing cycle |
| 54 | Scroll function (browse through for breathing cycles that are available in the folder) |
| 55 | Check the raw text data |
| 56 | Reset individual breathing cycle view |
| 57 | Breathing cycle view (Airway pressure in cmH_2_O, airway flow in dL/s, volume in dL, the changes in airway pressure cmH_2_O/s and the changes in airway flow dL/s^2^) |
| 58 | Inspiratory breathing cycle view (Airway pressure in cmH_2_O, airway flow in dL/s, volume in dL, the changes in airway pressure cmH_2_O/s and the changes in airway flow dL/s^2^, Model based *E_rs_×V* in cmH_2_O, the changes in *E_rs_*×V in cmH_2_O/s, overlay of airway pressure for the past 5 breathing cycles) |
| 59 | Time-varying elastance during inspiration (*E_drs_* in cmH_2_O/l) |
| 60 | Parameter Display   - Breath cycle index: the breathing cycle number as recorded by the ventilato - Time: Time when breathing cycle occurs - Is Processed: is the breathing cycle data processed successfully - Applied Model: Used model in CURE Soft - PEEP: measured PEEP from airway pressure - *Stiffness*: Normalised area under the cure for time-varying elastance (*AUCE_drs_* in cmH_2_O/l) - Ers: Current breathing cycle respiratory elastance - Rrs: Current breathing cycle respiratory resistance - Eclinical: Dynamic elastance = Peak airway pressure – PEEP/ Tidal Volume. |
| 61 | Statistics (min/max/avg/std): Simple data statistic of a specific breathing cycle (Minimum, Maximum, average and standard deviation)   - Insp P (cmH­_2_O): Statistic for inspiratory airway pressure - Insp Q (dL/s): Statistic for inspiratory airway flow - Insp V (L): Statistic for inspiratory airway volume - Exp P (cmH­_2_O): Statistic for expiratory airway pressure - Exp Q (dL.s): Statistic for expiratory airway flow - Exp V (L): Statistic for expiratory airway volume |

**D. CURE Soft Model Testing with Mechanical Lung**

The CURE Soft model is tested using a mechanical lung (Michigan Instruments Dual Adult Test Lung) connected to Puritan Bennett 840 ventilator. The mechanical lung was set with elastance of 6 or 20 cmH_2_O/l. Then, then mechanical lung is ventilated at different ventilator settings as shown in table below. The respiratory elastance estimated using CURE Soft is median 6.44 cmH_2_O/l and 22.22 cmH_2_O/l (within 10% deviation from the setting). Equally, the model fitting error is less than 5%, showing relatively good model estimation).

**Case 1**: Mechanical Lung Elastance at 6 cmH_2_O/l

| **Ventilator Settings** | | | | | **Curve fitting error for each breath** | |
| --- | --- | --- | --- | --- | --- | --- |
| **Mode** | **PEEP**  **(cmH_2_O)** | **PIP***  **(cmH_2_O)** | **Vt* (mL)** | **Flow profile** | **Median [IQR]** | **No. of Breathing Cycle** |
| Bi-Level | 10 | 15 | - | - | 0.44 [0.40 – 0.49] | 38 |
| Bi-Level | 10 | 20 | - | - | 0.47 [0.44 – 0.51] | 38 |
| Bi-Level | 15 | 20 | - | - | 0.35 [0.31 – 0.38] | 26 |
| SIMV-PC | 10 | 20 | - | - | 0.47 [0.45 – 0.51] | 31 |
| SIMV-PC | 15 | 25 | - | - | 0.38 [0.34 – 0.40] | 29 |
| SIMV-PC | 20 | 30 | - | - | 0.31 [0.29 – 0.34] | 27 |
| SIMV-VC | 10 | - | 300 | Ramp | 1.85 [1.70 – 1.93] | 35 |
| SIMV-VC | 10 | - | 300 | Square | 2.33 [2.22 – 2.42] | 22 |
| SIMV-VC | 10 | - | 600 | Ramp | 1.30 [1.24 – 1.36] | 33 |
| SIMV-VC | 10 | - | 600 | Square | 0.95 [0.65 – 1.08] | 26 |
| SIMV-VC | 20 | - | 300 | Ramp | 1.06 [0.99 – 1.10] | 23 |
| SIMV-VC | 20 | - | 300 | Square | 1.49 [0.72 – 1.85] | 35 |
| SIMV-VC | 20 | - | 600 | Ramp | 0.83 [0.79 – 0.87] | 28 |
| SIMV-VC | 20 | - | 600 | Square | 0.27 [0.22 – 0.36] | 29 |

Bi-Level - Bi-level pressure control model; SIMV-PC - Synchronous intermittent mandatory ventilation, pressure control model; SIMV-VC - Synchronous intermittent mandatory ventilation, volume control model; PEEP - Positive end expiratory pressure; PIP - Peak inspiratory pressure; Vt - Tidal volume

**Case 2**: Mechanical Lung Elastance at 20 cmH_2_O/l

| **Ventilator Settings** | | | | | **Curve fitting error for each breath** | |
| --- | --- | --- | --- | --- | --- | --- |
| **Mode** | **PEEP**  **(cmH_2_O)** | **PIP**  **(cmH_2_O)** | **Vt**  **(mL)** | **Flow profile** | **Median [IQR]** | **No. of Breathing Cycle** |
| Bi-Level | 10 | 15 | - | - | 0.70 [0.62 – 0.73] | 23 |
| Bi-Level | 10 | 20 | - | - | 1.01 [0.93 – 1.22] | 22 |
| Bi-Level | 15 | 20 | - | - | 0.48 [0.43 – 0.54] | 24 |
| SIMV-PC | 10 | 25 | - | - | 1.65 [1.38 – 1.78] | 24 |
| SIMV-PC | 15 | 30 | - | - | 1.27 [1.09 – 1.49] | 21 |
| SIMV-PC | 20 | 35 | - | - | 1.13 [0.97 – 1.29] | 22 |
| SIMV VC | 10 | - | 300 | Ramp | 1.13 [1.05 – 1.31] | 27 |
| SIMV VC | 10 | - | 300 | Square | 4.34 [3.83 – 4.56] | 22 |
| SIMV VC | 10 | - | 600 | Square | 0.67 [0.47 – 0.83] | 26 |
| SIMV VC | 20 | - | 300 | Ramp | 0.83 [0.75 – 0.92] | 25 |
| SIMV VC | 20 | - | 300 | Square | 1.33 [1.21 – 1.46] | 25 |
| SIMV VC | 20 | - | 600 | Ramp | 0.60 [0.57 – 0.68] | 27 |
| SIMV VC | 20 | - | 600 | Square | 0.44 [0.32 – 0.51] | 21 |

Bi-Level - Bi-level pressure control model; SIMV-PC - Synchronous intermittent mandatory ventilation, pressure control model; SIMV-VC - Synchronous intermittent mandatory ventilation, volume control model; PEEP - Positive end expiratory pressure; PIP - Peak inspiratory pressure; Vt - Tidal volume

**E. Phase Lag due to Moving Average**

In this version of CURE Soft, a default of 60 breaths moving average was used (black line). Resulting in a phase lag. The CURE Soft can be modified to different moving average depending on user requirements. The following shows moving average of 10 breaths, 30 breaths and 60 breaths, where the advantage and disadvantages are discussed.

| **10 Breathing cycles.**  The smooth *stiffness* aligns with breath-to-breath *stiffness* relatively close and It is a better representation in this patient example, due to the patient’s condition in response to the changes in mechanical ventilation settings.  It is relatively noisy when the patient is spontaneously breathing | 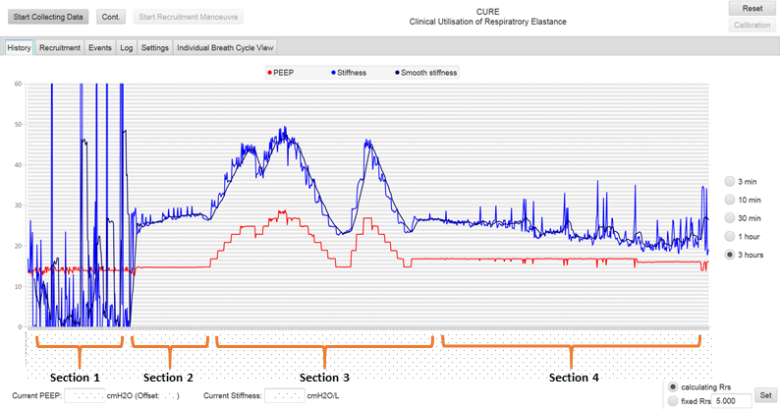 |
| --- | --- |
| **30 Breathing cycles.**  A small Phase lag is present when 30 breathing cycles is used.  A balance between 10 and 60 breathing cycles moving average. | 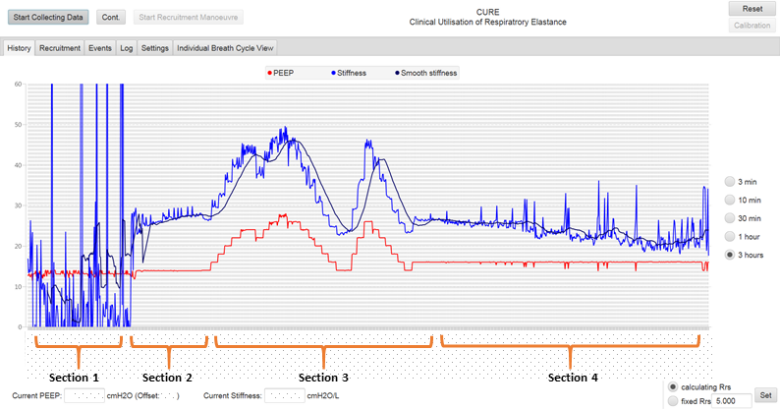 |
| **60 Breathing cycles.**  A larger phase lag is present when 60 breathing cycles is used.  It is more stable representation of patient’s condition when there is no sudden change in patient’s respiratory *stiffness*. | 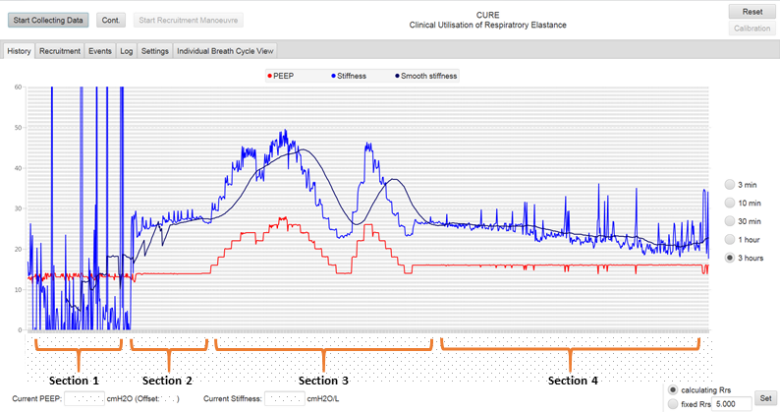 |
